# Adaptive Fisher method detects dense and sparse signals in association analysis of SNV sets

**DOI:** 10.1186/s12920-020-0684-3

**Published:** 2020-04-03

**Authors:** Xiaoyu Cai, Lo-Bin Chang, Jordan Potter, Chi Song

**Affiliations:** 1 Department of Statistics, The Ohio State University, 1948 Neil Ave., Columbus, OH 43210, US; 2 Department of Mathematics and Statistics, Kenyon College, 201 N College Rd., Gambier, Ohio 43022, US; 3 College of Public Health, Division of Biostatistics, The Ohio State University, 1841 Neil Ave., 208E Cunz Hall, Columbus, OH 43210, US

**Keywords:** Genome-wide association study, Adaptive fisher, Rare variants, Common variants, Dense signal, Sparse signal, Combine *p*-values

## Abstract

**Background:**

With the development of next generation sequencing (NGS) technology and genotype imputation methods, statistical methods have been proposed to test a set of genomic variants together to detect if any of them is associated with the phenotype or disease. In practice, within the set, there is an unknown proportion of variants truly causal or associated with the disease. There is a demand for statistical methods with high power in both dense and sparse scenarios, where the proportion of causal or associated variants is large or small respectively.

**Results:**

We propose a new association test – weighted Adaptive Fisher (wAF) that can adapt to both dense and sparse scenarios by adding weights to the Adaptive Fisher (AF) method we developed before. Using simulation, we show that wAF enjoys comparable or better power to popular methods such as sequence kernel association tests (SKAT and SKAT-O) and adaptive SPU (aSPU) test. We apply wAF to a publicly available schizophrenia dataset, and successfully detect thirteen genes. Among them, three genes are supported by existing literature; six are plausible as they either relate to other neurological diseases or have relevant biological functions.

**Conclusions:**

The proposed wAF method is a powerful disease-variants association test in both dense and sparse scenarios. Both simulation studies and real data analysis indicate the potential of wAF for new biological findings.

## Background

Single nucleotide variants (SNVs) are a type of chromosome variants where the DNA sequence of an individual is different from the reference genome on only one nucleotide. Before the era of next generation sequencing (NGS), SNP array technology was used to obtain the genotypes of common SNVs with minor allele frequencies (MAFs) larger than a certain cutoff (e.g. 1% or 5%, a.k.a single nucleotide polymorphisms or SNPs). Over the past decades, genome-wide association studies (GWASs) have been successfully conducted to discover many disease-associated common SNVs with relatively large MAFs [1, 2]. Despite the success of GWAS, the common SNVs detected through this procedure sometimes account for only a small proportion of the heritability, which is known as the problem of “missing heritability” [3]. This problem promotes the researchers to seek heritability outside of the controversial common disease-common variant hypothesis, which is the fundamental of GWAS based on common SNVs, but to seek “missing heritability” in rare SNVs [4]. Rare SNVs (a.k.a rare variants) are SNVs with low MAFs (often <1*%* or <5*%*). Compared to common SNVs, the number of rare SNVs is much larger, and their locations on the human genome are often unknown before genotyping all the study samples, which makes DNA hybridization-based genotyping technology (e.g. SNP array) less useful in genotyping rare SNVs. Thanks to the advent of NGS, researchers now are enabled to reliably measure rare SNVs. Furthermore, because of the development of fast imputation tools [5] and the 1000 Genomes project [6], rare SNVs can be imputed for old GWASs where only common SNVs were measured. This helps recycle and add value to the numerous GWASs that are conducted for many complex human diseases and are available on public domain.

However, the technological advancement in genotyping of rare SNVs also presents several statistical challenges for the association analysis method development. First, because of the small MAFs of the rare variants, the statistical power of traditional association methods are very low when applied to detect the association between rare variants and the disease outcome. Second, because the number of SNVs including both common and rare variants is significantly larger than the number of common variants (often more than 100 times larger), the multiple comparison issue is more severe [7]. Therefore, it would be powerless if association analysis were performed on each single SNV separately. A commonly used solution to these issues was to perform the association analysis on SNV sets, where multiple SNVs were grouped together based on their locations on the genome. SNVs on or close to a gene are often grouped together into one SNV set. However, the traditional statistical testing methods such as the score test or likelihood ratio test used in multivariate generalized linear models (GLMs) are not powerful enough when many variants are included in the SNV set. As shown by Fan [8], the tests based on *χ*^2^ distribution will have no power when the signal is weak or rare as the degree of freedom increases. To solve this problem, three categories of approaches have been proposed, all of them essentially reduced the degree of freedom in some way to boost the statistical power.

The first category is burden tests, which collapse rare variants into genetic burdens, then test the effects of the genetic burden. CAST [9], CMC [10] and wSum [11] all belong to this category. By combining multiple rare variants into a single measurement of genetic burden, these methods essentially reduce the number of parameters to test down to one, which is equivalent to reducing the degree of freedom of the *χ*^2^ test statistic to one. Despite the popularity of this type of method, the traditional way of calculating genetic burden often ignores the fact that different variants may have opposite effects on the same outcome. Simply pooling or summing the variants together may cause the opposite effects to cancel out, therefore reduce the statistical power. A solution is to calculate genetic burden adaptively based on evidence provided by the data. For example, Price et al. [12] proposed to adjust MAF threshold for the pooling step based on data. Han and Pan [13] and Hoffmann et al. [14] proposed to adaptively choose the sign and magnitude of the weight in the collapsing step to calculate genetic burdens. TARV [7] can also be viewed as this type of method because it adaptively combines multiple variants into a “super variant” based on the strength of evidence provided by each single variant.

The second category of methods is quadratic tests which often base on testing variance component in mixed effect models. The well-known SKAT [15] belongs to this category. By assuming the effect of each variant to be random, SKAT tests whether the variance of the random effects is zero. The test statistic can be approximated by a *χ*^2^ distribution with a degree of freedom much smaller than that in the likelihood ratio test (or Rao’s score test) in fixed effect models. SKAT can also test non-linear effects by adopting an arbitrary kernel matrix. SKAT was also extended to accommodate multiple candidate kernels [16], to jointly test rare and common variants [17], and to apply on family data [18]. Some other popular methods, such as C-alpha [19] and SSU [20] can be viewed as special cases of SKAT.

The third category is functional analysis. Because the genomic variants within the same gene are often highly correlated due to linkage disequilibrium (LD), this category of methods treat them as discrete realizations of a hidden continuous function on the genome. Both the variants and their coefficients can then be decomposed in the functional space. Since the number of functional bases used is generally smaller than the number of variants, this is equivalent to a dimensional reduction method which also reduces the degree of freedom of the association test. Different methods under this category have been proposed utilizing different basis including functional principal component basis [21], B-spline basis [22, 23], and Fourier basis [23].

In addition to these three categories of methods, efforts have also been made to combine multiple testing methods into one single test. For example, the popular SKAT-O [24] is a combination of variance component test (SKAT) and burden test. Similarly, Derkach et al. [25] proposed to combine variance component test and burden test using Fisher’s method or minimal *P*-value.

It should be noted that the power of aforementioned methods relies on the proportion of variants which truly associate with the disease outcome. Under the alternative hypothesis – when the null hypothesis of no association is not true, all three types of methods assume that every SNV included in the test has some nonzero effect more or less. Specifically, burden tests assume the effects of the variants are proportional to each other, with the proportion predefined by the weights used to calculate the genetic burden; variance component tests assume the random effects of the combined variants share a common variance component, if the component is not zero implies all the random effects are nonzero; and the functional analysis based methods test whether any functional basis (a weighted sum of variants) has a nonzero effect, which in turn implies nonzero effects for all or most of the variants. The type I error of these methods is not affected by violation of this assumption of the alternative hypothesis, which does not undermine their validity. However, under the alternative hypothesis where some effects are nonzero (especially when only a small proportion of variants have nonzero effects), the statistical power of these tests will be suboptimal. Therefore there is a demand for statistical methods that can adapt to the proportion of variants with nonzero effects. For the ease of discussion, we call the scenario where this proportion is large as the dense scenario, and call the scenario where this proportion is small as the sparse scenario. For this purpose, Pan et al. [26] proposed an adaptive test named aSPU which has strong statistical power in both the dense and sparse scenarios. This aSPU can also be viewed as a combination of SKAT (with linear kernel) and other tests including burden test. Barnett and Lin [27] suggested that Higher Criticism (HC) can be another potential powerful test that can adaptively detect both dense and sparse signals. Previously, we proposed Adaptive Fisher (AF) method [28] and illustrated in simulation that AF is a very powerful method to detect the mixture distribution in both dense and sparse scenarios, and it can be much more powerful than HC with finite sample. Therefore, we propose to use AF to detect disease-associated SNV sets, and compare to existing methods in the following sections.

## Methods

Suppose a trait for *n* independent subjects ***Y***=(*Y*_*i*1_,...,*Y*_*in*_)^*T*^ are observed. ***G***_*i*_=(*G*_*i*1_,...,*G*_*iK*_)^*T*^ denotes the genotypes of *K* SNVs in a chromosomal region (e.g. a gene) for subject *i*, where *G*_*ik*_=0,1,2 represents the number of minor alleles at locus *k* of subject *i*. We model the association between the trait and SNVs with the following generalized linear model

1 $$  h\Big(E(Y_{i})\Big)=\beta_{0}+\sum_{k=1}^{K} \beta_{k} G_{ik},  $$

where ***β***=(*β*_1_,...,*β*_*K*_)^*T*^ is the vector of SNV effects. *h*(·) is taken as the logit link function for binary traits (e.g. diseased or nondiseased) or as the identity link function for continuous traits (e.g. blood pressure, height, etc.). If *J* covariates ***C***_*i*_=(*C*_*i*1_,...,*C*_*iJ*_)^*T*^, *i*=1,2,...,*n* are also observed for each subject, denoting their effects by ***α***=(*α*_1_,...,*α*_*J*_)^*T*^, the model can be extended as

2 $$  h\Big(E(Y_{i})\Big)=\beta_{0}+\sum_{k=1}^{K} \beta_{k} G_{ik}+\sum_{j=1}^{J}\alpha_{j} C_{ij}.  $$

Determining whether there is an association between the trait and any SNV is equivalent to testing the following hypotheses,

3 $$  H_{0}: \boldsymbol{\beta}=\boldsymbol{0} \quad \text{versus} \quad H_{1}: \boldsymbol{\beta} \neq \boldsymbol{0}.  $$

The proposed adaptive fisher tests involve the score statistics ***U***=(*U*_1_,...,*U*_*K*_)^*T*^. For model (1),

4 $$  \boldsymbol{U}=\sum_{i=1}^{n} (Y_{i}-\bar{Y})\boldsymbol{G}_{i},  $$

and its estimated covariance matrix under *H*_0_ is given by

5 $$  \boldsymbol{V}=\widehat{Cov}(U|H_{0})=\bar{Y}(1-\bar{Y})\sum_{i=1}^{n} \left(\boldsymbol{G}_{i}-\bar{\boldsymbol{G}}\right)\left(\boldsymbol{G}_{i}-\bar{\boldsymbol{G}}\right)^{T},  $$

for binary traits, and

6 $$  \boldsymbol{V}=\widehat{Cov}(U|H_{0})=\hat{\sigma}_{1}^{2} \sum_{i=1}^{n} (\boldsymbol{G}_{i}-\bar{\boldsymbol{G}})(\boldsymbol{G}_{i}-\bar{\boldsymbol{G}})^{T}.  $$

for continuous traits, where $\bar {Y}=\frac {1}{n} \sum _{i=1}^{n} Y_{i}$, $\hat {\sigma }_{1}^{2}=\frac {1}{n-1} \sum _{i=1}^{n} (Y_{i}-\bar {Y})^{2}$ and $\bar {\boldsymbol {G}}=(\bar {G}_{\cdot 1},..., \bar {G}_{\cdot K})^{T}$ with $\bar {G}_{\cdot k}=\frac {1}{n} \sum _{i=1}^{n} G_{ik}$. For model (2),

7 $$  \boldsymbol{U}=\sum_{i=1}^{n} \left(Y_{i}-\hat{\mu}_{Y_{i}}\right)\left(\boldsymbol{G}_{i}-\hat{\boldsymbol{G}_{i}}\right),  $$

for binary traits,

8 $$  \boldsymbol{V}=\widehat{Cov}(U|H_{0})=\hat{\sigma}_{2}^{2} \sum_{i=1}^{n} \left(\boldsymbol{G}_{i}-\hat{\boldsymbol{G}_{i}}\right)\left(\boldsymbol{G}_{i}-\hat{\boldsymbol{G}_{i}}\right)^{T},  $$

and for continuous traits,

9 $$  \boldsymbol{V}=\widehat{Cov}(U|H_{0})=\hat{\sigma}_{3}^{2} \sum_{i=1}^{n} \left(\boldsymbol{G}_{i}-\hat{\boldsymbol{G}_{i}}\right)\left(\boldsymbol{G}_{i}-\hat{\boldsymbol{G}_{i}}\right)^{T},  $$

where $\hat {\mu }_{Y_{i}}=h^{-1}\left (\hat {\beta }_{0}+\sum _{j=1}^{J}\hat {\alpha }_{j} C_{ij}\right)$ with $\hat {\beta }_{0}$ and $\hat {\alpha }_{j}, \ j=1,2,...,J$ being the maximum likelihood estimators, $\hat {\boldsymbol {G}_{i}}=\left (\hat {G}_{i1},...,\hat {G}_{ik}\right)^{T}$ with $\hat {G}_{ik}$ being the predictive value of *G*_*ik*_ from a linear regression model with covariates as predictors. $\hat {\sigma }_{2}^{2}=\frac {1}{n} \sum _{i=1}^{n} \hat {\mu }_{Y_{i}}(1-\hat {\mu }_{Y_{i}})$, $\hat {\sigma }_{3}^{2}=\frac {1}{n-1} \sum _{i=1}^{n} (e_{i}-\bar {e})^{2}$ with $e_{i}=Y_{i}-\hat {\mu }_{Y_{i}}, \ i=1,2,...,n$ and $\bar {e}=\frac {1}{n} \sum _{i=1}^{n} e_{i}$.

### Adaptive fisher method

Let the standardized score statistics be $\tilde {U}_{k}=U_{k}/\sqrt {V_{kk}}$, where *V*_*kk*_ is the *k*^th^ diagonal element of ***V***. If *β*_*k*_ is tested marginally, the *P*-value for this marginal score test is $p_{k}=2\left [1-\Phi \left (|\tilde {U_{k}}|\right)\right ]$, *k*=1,2,...,*K*, as $\tilde {U}_{k}$ is asymptotically *N*(0,1) distributed under *H*_0_. Let

10 $$  R_{k}=-\log \ p_{k}.  $$

Order *R*’s in descending order *R*_(1)_≥⋯≥*R*_(*K*)_. Let ***S***=(*S*_1_,...,*S*_*K*_)^*T*^ be the partial sums of *R*_(1)_,...,*R*_(*K*)_,

11 $$  S_{k}=\sum_{l=1}^{k} R_{(l)}.  $$

For each *S*_*k*_, *k*=1,2,...,*K*, we calculate its *P*-value by

12 $$  P_{s_{k}}=\text{Pr}(S_{k} \geq s_{k}),  $$

where *s*_*k*_ is be observed value of *S*_*k*_. The AF test is based on the AF statistic below

13 $$  T_{\text{AF}}=\min_{1 \leq k \leq K} P_{s_{k}}.  $$

### Weighted adaptive fisher method

SNVs can be weighed differently when taking the partial sums. Suppose ***w***=(*w*_1_,...,*w*_*K*_)^*T*^ are weights of the *K* SNVs in a genetic region. Define

14 $$  X_{k}=w_{k} R_{k}.  $$

Order *X*_1_,...,*X*_*K*_ in descending order *X*_(1)_≥⋯≥*X*_(*K*)_. Let $\boldsymbol {S^{*}}=(S^{*}_{1},...,S^{*}_{K})^{T}$ be the partial sums of *X*_(1)_,...,*X*_(*K*)_,

15 $$  S^{*}_{k}=\sum_{l=1}^{k} X_{(l)}.  $$

Similar to (12), the *P*-value of $s^{*}_{k}$ (observed value of $s^{*}_{k}$), $P_{s^{*}_{k}}= \text {Pr}(S^{*}_{k} \geq s^{*}_{k})$, and the weighted AF (wAF) statistic is defined by

16 $$  T_{\text{wAF}}=\min_{1 \leq k \leq K} P_{s^{*}_{k}}.  $$

### Directed wAF method

We use two-sided *P*-values of marginal tests to construct AF and wAF methods in the above sections. However, when all or most of the causal variants have effects of the same direction, combining one-sided *P*-values using the same strategy may have higher statistical power. Therefore, we propose a directed version of wAF, noted as wAF _d_. Let $p_{k}^{+}=1-\Phi (\tilde {U_{k}})$, *k*=1,2,...,*K* be the one-sided *P*-values of testing whether the variants are risk factors (i.e. testing *H*_0_:*β*_*k*_=0 versus *H*_1_:*β*_*k*_>0), and $p_{k}^{-}=\Phi (\tilde {U_{k}})$ be the one-sided *P*-values of testing whether the variants are protective (i.e. testing *H*_0_:*β*_*k*_=0 versus *H*_1_:*β*_*k*_<0). We first combine ***p***=(*p*_1_,...,*p*_*k*_), $\boldsymbol {p}^{+}=\left (p_{1}^{+},..., p_{k}^{+}\right)$ and $\boldsymbol {p}^{-}=\left (p_{1}^{-},..., p_{k}^{-}\right)$ following Eqs. 10 and (14)-(16) to obtain *T*_wAF_, *T*_wAF+_ and *T*_wAF-_ respectively. Then, we define the minimal of three as the wAF _d_ statistic, which is

17 $$  T_{\text{wAF\(_{\mathrm{d}}\)}}=\min \{ T_{\text{wAF}}, T_{\text{wAF+}}, T_{\text{wAF-}}\}.  $$

### Computation

We use the following procedure to access $P_{s_{k}}$ ($P_{s^{*}_{k}}$) in (12) and find the null distributions of *T*_AF_ in (13) (*T*_wAF_ in (16)). Here the weighted method for model (1) is used as an example. The unweighted method can be regarded as a special case with all weights being equal.

- Calculate the residuals $e_{i}=Y_{i}-\bar {Y}$, *i*=1,2,...,*n*.
- Permute *e*_*i*_’s for a large number *B* times to obtain $\boldsymbol {e}^{(b)}=\left (e^{(b)}_{1},...,e^{(b)}_{n}\right)^{T}$, *b*=1,2,...,*B*, where $\left (e^{(b)}_{1},...,e^{(b)}_{n}\right)^{T}$ is a permutation of ***e***^(0)^=(*e*_1_,...,*e*_*n*_)^*T*^.
- For each ***e***^(*b*)^, calculate $\boldsymbol {U}^{(b)}=\left (U^{(b)}_{1},...,U^{(b)}_{K}\right)^{T}=\sum _{i=1}^{n} e^{(b)}_{i} \boldsymbol {G}_{i}$ and $\boldsymbol {p}^{(b)}=\left (p^{(b)}_{1},...,p^{(b)}_{K}\right)^{T}$ with $p^{(b)}_{k}=2\left [1-\Phi \left (\left |U^{(b)}_{k}/\sqrt {V_{kk}}\right |\right)\right ]$. Then follow Eqs. 10, (14) and (15) to get $\boldsymbol {S}^{*(b)}=\left (S^{*(b)}_{1},...,S^{*(b)}_{K}\right)^{T}$, *b*=0,1,2,..,*B*.
- For a fixed *b*^∗^∈{0,1,2,...*B*},
  $$P^{(b^{*})}_{S^{*}_{k}}=\frac{1}{B+1} \sum_{b=0}^{B} \mathbb{I} \left\{S^{*(b)}_{k} \geq S^{*(b^{*})}_{k}\right\}.$$
- For each ***S***^∗(*b*)^, $T_{\text {wAF}}^{(b)}=\min _{1 \leq k \leq K} P_{S^{*}_{k}}^{(b)}$, *b*=0,1,2,...,*B*.
- The *P*-value of wAF test can be approximated by
  $${}\widehat{\text{Pr}}\left\{\left. T_{\text{wAF}} \leq T_{\text{wAF}}^{(0)}\right|H_{0}\right\}=\frac{1}{B+1} \sum_{b=1}^{B} \mathbb{I}\left\{ T_{\text{wAF}}^{(b)} \leq T_{\text{wAF}}^{(0)}\right\},$$
  where $T_{\text {wAF}}^{(0)}=\min _{1 \leq k \leq K} P_{S^{*}_{k}}^{(0)}$ is the observed value of the wAF statistic and $\mathbb {I}(\cdot)$ is the indicator function.

Note that the *P*-value of $T_{\text {wAF\(_{\mathrm {d}}\)}}$ can be assessed using a similar permutation procedure.

## Results

In this section, we evaluate our wAF and wAF _d_ methods by both simulation studies and real-data application. In simulation studies, we compare our methods with SKAT, SKAT-O, aSPU and Min-P (which takes the minimal *P*-value of all the combined variants as the test statistic). In real-data application, we use the Genome-Wide Association Study of Schizophrenia (SCZ) data provided by Genetic Association Information Network (GAIN), which is publicly available in the database of Genotypes and Phenotypes (dbGaP).

### Simulation studies

Simulation studies are conducted under both dense and sparse scenarios to compare various methods. Genotypes ***G***_*i*_=(*G*_*i*1_,...,*G*_*iK*_)^*T*^, *i*=1,2,...,*n* are simulated in a similar manner to the framework of Pan et al. [26], by the following steps.

- Generate $\phantom {\dot {i}\!}\boldsymbol {Z_{1}}=(Z_{11},...,Z_{1K})^{T}$ and ***Z***_***2***_=(*Z*_21_,...,*Z*_2*K*_)^*T*^ independently from a multivariate normal distribution *N*(***0***,***A***). ***A*** has a first-order autoregressive (AR(1)) covariance structure with the (*k*,*k*^′^)^th^ element $A_{kk'}=c^{|k-k'|}\phantom {\dot {i}\!}$. *c* is chosen to be 0.9 to give close loci a higher correlation and distant loci a lower correlation.
- Randomly sample MAFs by first generating log(MAF)’s from *U*(log(0.001), log(0.5)) and then exponentiating them back to MAFs.^1^ Set $G_{ik}=\mathbb {I}(\Phi (Z_{1k}) \leq \text {MAF}_{k})+\mathbb {I}(\Phi (Z_{2k}) \leq \text {MAF}_{k})$, *k*=1,...,*K*.
- Repeat step 1 and 2 *n* times to generate genotypes for all subjects.

We randomly sample *πK* genotype effects among ***β***=(*β*_1_,...,*β*_*K*_)^*T*^ to be nonzero, whose values are sampled from a uniform distribution within [−*δ*,*δ*], while keep the other (1−*π*)*K* effects zeros. Trait of *n*=1,000 subjects are generated from model (1).

The weights of wAF and wAF _d_ tests are chosen to be $w_{k}=\sqrt {\text {MAF}_{k}(1-\text {MAF}_{k})}$, *k*=1,2,..,*K*. The weights of SKAT and SKAT-O are chosen to be flat with *w*_*k*_=1, *k*=1,2,...,*K*, so that SKAT is equivalent to SSU [15]. The significance level is set to be 0.05 for every test. All simulation results are based on 5,000 replicates.

#### Binary traits

**Fig. 1:**
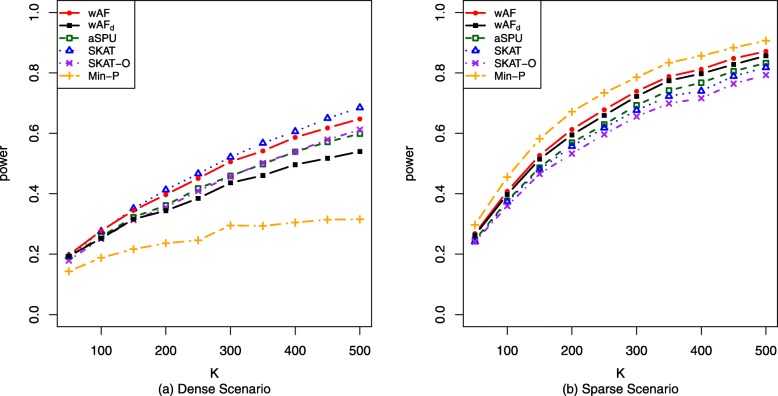
Power curves for binary trait. Comparison of empirical powers for binary trait. **a** Power against varying number of loci *K* in the dense scenario with effect proportion *π*=20*%* and effect size *δ*=0.2. *K*∈{50,100,...,450,500}. **b** Power against varying number of loci *K* in the sparse scenario with effect proportion *π*=2*%* and effect size *δ*=1. *K*∈{50,100,...,450,500}

When generating binary trait, *h*(·) is taken to be the logit link function. We increase the number of SNVs, *K*, from 50 to 500 with an increment 50, while holding the effect proportion *π* and the effect size *δ* constant. For the dense scenario, *π*=20*%* and *δ*=0.2. For the sparse scenario, *π*=2*%* and *δ*=1. Figure 1 shows that wAF test results in large powers for both dense and sparse scenarios. Specifically, in the dense scenario, wAF and SKAT have the highest power. SKAT-O and aSPU are slightly less powerful than SKAT and wAF. wAF _d_ follows behind with almost the same for small *K* and slightly inferior performance for large *K*. Min-P, on the other hand, is much less powerful than the other methods. For the sparse scenario, Min-P is the most powerful method. Our wAF has the second highest power, which is about 5% higher than the other methods including SKAT, SKAT-O and aSPU. wAF _d_ has the third best performance, following tightly after wAF. For all these compared methods, the type I errors are well-controlled empirically as shown in the Additional file 1: Table S1.

#### Continuous traits

**Fig. 2:**
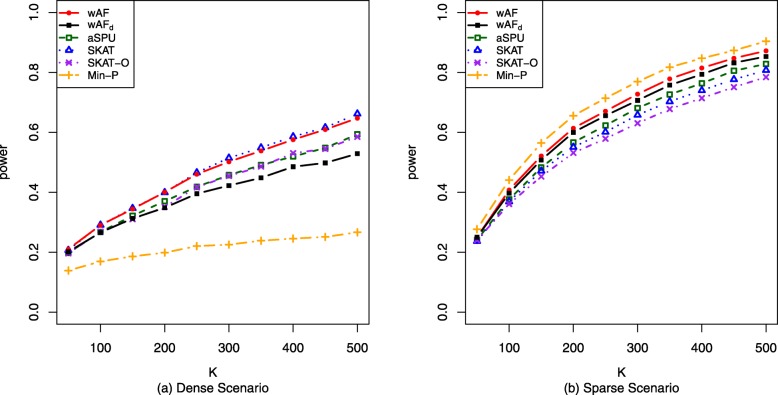
Power curves for continuous trait. Comparison of empirical powers for continuous trait. **a** Power against varying number of loci *K* in the dense scenario with effect proportion *π*=20*%* and effect size *δ*=0.1. *K*∈{50,100,...,450,500}. **b** Power against varying number of loci *K* in the sparse scenario with effect proportion *π*=2*%* and effect size *δ*=0.5. *K*∈{50,100,...,450,500}

When generating continuous trait, *h*(·) is taken to be the identity link function and random errors are standard normal random variables. Again, *K* is increased from 50 to 500 with an increment 50, while *π* and *δ* are held constants. For the dense scenario, *π*=20*%* and *δ*=0.1. For the sparse scenario, *π*=2*%* and *δ*=0.5. Based on power curves in Fig. 2, wAF test performs relatively well for both dense and sparse scenarios, similar to what we have seen in the binary traits. In dense scenario, wAF and SKAT enjoy the highest power, which is slightly better than aSPU, SKAT-O and wAF _d_, and much better than Min-P. Whereas in the sparse scenario, Min-P is the most powerful method, seconded by wAF. wAF _d_ has slightly less power than wAF, but has higher power than aSPU, SKAT and SKAT-O. Similar to the binary traits, all type I errors are well-controlled empirically.

We also consider two other cases where 1) all SNV effects are of the same direction; 2) all variants are rare variants (RVs). In the first case, nonzero *β*_*k*_’s are sampled from *U*[0,*δ*] distribution. The result shows that wAF _d_ has large powers in both dense and sparse scenarios. wAF has almost the same high power with wAF _d_ in the sparse scenario. In the second case, MAF’s are generated from *U*(0.001,0.01). wAF and wAF _d_ work well especially in the sparse scenario. Power curves for these two cases are shown in Additional file 1: Figure S1–3.

### Real data application

To further evaluate the performance of our methods, we apply wAF and wAF _d_ on European-American subjects from GAIN SCZ data. 2,548 subjects are selected after quality control, including 1,170 cases and 1,378 controls. Genotypes are imputed using Michigan Imputation Server [5] to UCSC Human Genome build hg19. We focus our analysis on variants that are within genes and their flanking regions (5,000 bp upstream and downstream). The analysis is performed on 13,993,898 variants located on 18,296 autosomal genes.

We apply wAF and wAF _d_ methods based on model (1) for each gene, with disease status as the outcome and genotypes of SNVs in this gene as predictors. *P*-values are estimated using a similar step-up procedure as in Pan et al. [26] such that the data analysis can be more computationally efficient. We firstly scan all genes with *B*=100 permutations. For each gene, if the estimated *P*-value is smaller than 5/*B*, we continue with *B*=1,000; otherwise, we stop the procedure for this gene and record the estimated *P*-value. Each round *B* is increased to 10 times of its current value for those significant genes until no gene has a *P*-value smaller than 5/*B*.

Table 1 lists the ten most significant genes detected by either wAF or wAF _d_. FAM69A has the smallest *P*-value by both methods. Two transcriptome studies ([31, 32]) report FAM69A as a differentially expressed gene by affection status of SCZ. Wang et al. [33] identifies two SNPs (rs11164835 and rs12745968) within this gene that are associated with both SCZ and bipolar disorder (BD) by a genome-wide meta-analysis. Another gene in our list, HPGDS, is also mentioned as related to both diseases [34]. Besides, GTF2A1 is found associated with BD by Fries et al. [35]. Increasing evidence of SCZ and BD being closely related ([36], [37]) suggests GTF2A1 might be a candidate associated gene with SCZ.

**Table 1:** Summary of the Most Significant Genes in the GAIN Schizophrenia Data Analysis

Gene	wAF	wAF _*d*_	aSPU	SKAT	SKAT-O	Related Disease	Function
FAM69A	1.20 ×10^−5^	4.00 ×10^−5^	1.70 ×10^−5^	6.31 ×10^−6^	6.41 ×10^−6^	SCZ [33]	Protein binding.
						MS [39]	
						Parkinson’s Disease [29]	
NUDT12	6.00 ×10^−5^	5.99 ×10^−3^	4.80 ×10^−5^	4.29 ×10^−3^	6.58 ×10^−3^	Depressive Symptoms [43]	Regulates the concentrations of individual
							nucleotides and of nucleotide ratios.
RPL5	6.00 ×10^−5^	9.00 ×10^−5^	1.00 ×10^−4^	3.57 ×10^−5^	2.76 ×10^−5^	MS [39]	Ribosomal protein, binds 5s RNA.
HPGDS	8.00 ×10^−5^	6.00 ×10^−4^	1.50 ×10^−4^	5.16 ×10^−5^	7.89 ×10^−5^	SCZ [34]	PGH2 to PGD2 conversion enzyme.
SMARCAD1	1.00 ×10^−4^	1.30 ×10^−4^	1.10 ×10^−4^	6.06 ×10^−5^	1.53 ×10^−4^		Heterochromatin organization restoration
							epigenetic pattern propagation.
GTF2A1	1.20 ×10^−4^	1.00 ×10^−3^	1.70 ×10^−4^	9.90 ×10^−5^	9.98 ×10^−5^	BD [35]	Transcriptional activation.
NRN1L	1.20 ×10^−4^	3.50 ×10^−4^	6.00 ×10^−4^	2.04 ×10^−4^	2.63 ×10^−4^	Psychiatric Diseases[44]	Neurite growth, neuronal survival.
CERCAM	1.40 ×10^−4^	6.00 ×10^−4^	1.30 ×10^−4^	1.72 ×10^−1^	1.80 ×10^−1^		Probable cell adhesion protein.
SLC35A5	1.80 ×10^−4^	5.99 ×10^−3^	2.30 ×10^−3^	4.16 ×10^−4^	3.32 ×10^−4^	Autistic Disorder[30]	Nucleoside-sugar transporter.
STRA13	2.00 ×10^−4^	9.00 ×10^−5^	9.00 ×10^−5^	9.81 ×10^−5^	1.07 ×10^−4^	SCZ [46]	Mitotic progression and chromosome segregation.
ESRP2	2.90 ×10^−4^	2.60 ×10^−4^	4.30 ×10^−4^	1.85 ×10^−4^	1.97 ×10^−4^		An epithelial cell-type-specific splicing regulator.
LCAT	6.00 ×10^−4^	6.00 ×10^−4^	3.10 ×10^−4^	9.80 ×10^−4^	1.08 ×10^−3^		Enzyme in the extracellular metabolism.
KIAA1024L	1.00 ×10^−3^	6.00 ×10^−4^	1.00 ×10^−3^	8.76 ×10^−4^	6.66 ×10^−4^		

Gene RPL5 is the third significant by wAF and the second significant by wAF _d_. RPL5 is identified by International Multiple Sclerosis Genetics Consortium (IMSGC) [38] and Rubio et al. [39] as a risk allele for multiple sclerosis (MS), an autoimmune disease which often causes neurological disability. Considering the genetic pleiotropy between SCZ and MS [40], RPL5 is a plausible gene that associates with SCZ. Furthermore, 21 SNPs are identified as positively associated with MS by IMSGC at the GFI-EVI5-RPL5-FAM69A locus. Associations between this region and MS are further replicated in independent studies among different populations [41, 42]. This may shed light on understanding the similarities and differences among SCZ, BD and MS.

For the other genes that we detect, Hek et al. [43] reports that SNP rs161645 near NUDT12 is associated with depressive symptoms; NRN1L expresses predominantly in the nervous system [44] and may play a role in psychiatric diseases [45]; and STRA13 may have an effect on SCZ by influencing gene CHRNA7 [46].

Among the thirteen genes listed in Table 1, FAM69A, HPGDS and STRA13 are previously found associated with SCZ by other researches; five genes (NUDT12, RPL5, GTF2A1, NRN1L and SLC35A5) are reported to be related to neurological diseases other than SCZ; NRN1L and CERCAM are plausible in terms of gene function.

We also compare wAF and wAF _d_ with aSPU, SKAT and SKAT-O on these genes. It is noticeable that the five methods perform differently on gene CERCAM. wAF, wAF _d_ and aSPU attain *P*-values that reach 1×10^−4^ while SKAT and SKAT-O can only reach 1×10^−1^. After calculating the marginal *P*-values for each of the 228 variants on CERCAM, we find that only 1 variant has a *P*-value smaller than 1×10^−4^ while the other *P*-values are all larger than 0.01 (details can be found in Additional file 1: Figure S4). This again shows that wAF, wAF _d_ and aSPU are superior than SKAT and SKAT-O in the sparse scenario, which is consistent with results from our simulation studies.

In summary, most of the genes we detect are supported by existing literature. This demonstrates the potential of real-life impact of our wAF methods, especially considering that we only used 2,548 subjects and the fact that SCZ GWAS is known to be limited by the sample size, yielding results that are not significant until the sample size reached tens of thousands [47].

## Discussion

Based on the simulation and real data analysis results, we found wAF has high power in both dense and sparse scenarios. This is because we adaptively combine the marginal tests based on the strength of evidence. By sorting the marginal *P*-values in ascending order, we only combine the most relevant SNVs into the test. The selection of partial sums allows wAF to have its adaptiveness, as the number of variants combined into the test depends on the unknown proportion of variants that are truly causal or associated. Variants with less or no evidence will not contribute to the final test, which in turn will reduce noise in the test statistic. Therefore, wAF enjoys the comparable or better power in both scenarios.

As stated in the “Background” section, HC is another method that can be used to combine marginal tests of each variant. Although we did not explore the application of HC in SNV set analysis, Barnett et al. [48] proposed a generalized higher criticism (GHC) based on HC. They found that GHC was only powerful in sparse scenario but underperformed in dense scenario, and suggested that one may consider combining GHC and SKAT to boost power when we do not know which scenario the causal gene actually belongs to, which we believe is true for every real-life problem. This conclusion agrees with our previous findings about HC [28].

While comparing wAF and aSPU, we found that their test statistics can be written in the same general format. For both methods, we can think the test statistic as adaptively chosen from a set of weighted sums with different weights. The weighted sums in both methods can be written as $\sum _{k} v_{c}(\tilde {U}_{k}, G_{k})w(G_{k})f(\tilde {U}_{k})$, where $v_{c}(\tilde {U}_{k}, G_{k})$ is the *c*th adaptive weight function depends on the standardized score statistic and the genotype data for variant *k*, *w*(*G*_*k*_) is a non-adaptive weight only depends on the genotype data, and $f(\tilde {U}_{k})$ is a transformation of the standardized score statistic. We can show that for aSPU, $f(\tilde {U}_{k})=\tilde {U}_{k}$, *w*(*G*_*k*_)=sd(*G*_*k*_), and $v_{c}(\tilde {U}_{k}, G_{k})=[w(G_{k})f(\tilde {U}_{k})]^{(c-1)}$ for *c*∈{1,2,…,8,*∞*}; for wAF, $f(\tilde {U}_{k})=2[1-\Phi (|\tilde {U}_{k}|)]$, $w(G_{k})=\sqrt {\text {MAF}_{k}(1-\text {MAF}_{k})}\approx \text {sd}(G_{k})/\sqrt {2}$, and $v_{c}(\tilde {U}_{k}, G_{k})=\mathbb {I}\{w(G_{k})f(\tilde {U}_{k})\ge [w(G)f(\tilde {U})]_{(c)}\}$ for *c*∈{1,2,…,*K*}, where $\mathbb {I}\{\cdot \}$ is an indicator function and [·]_(*c*)_ denotes the *c*th largest order statistics of the quantity inside the bracket. By comparison, we can see that the major difference between aSPU and wAF is how we adaptively weigh the test statistic: aSPU creates the weight by raising the statistics to different powers, whereas wAF sequentially put a 0/1 weight based on the magnitude of the test statistics. This comparison also reveals that although not explicitly mentioned, aSPU also weighs different variants based on their MAFs using almost the same weight as we used in wAF.

Because permutation is needed for wAF, computational burden is a major weakness. To improve computation speed, we adopt the same strategy as Pan et al. [26] to run a hundred permutation first, then choose to increase the number of permutation only for those with small *P*-values. Theoretically, because sorting and order statistics are used in wAF, the computation complexity is higher than aSPU. Specifically, because wAF need sorting and cumulative summation, our complexity is higher than aSPU by an order of log*K*. In practice, because *K* is often fixed, the theoretical difference in computational complexity can be ignored.

## Conclusions

Association analysis of SNV sets becomes the standard analysis approach in GWAS when rare variants are genotyped or imputed in the dataset. However, when many SNVs are combined together into one omnibus test, the power of the statistical test often depends on the proportion of variants with nonzero effects and how these variants are combined. Most current methods (except aSPU) are not adaptive to this proportion and only applies to either the dense or sparse scenario. In this paper, we proposed a new adaptive method wAF as an alternative to aSPU with better or comparable power. The adaptiveness of wAF allows it to perform better than current available methods in both dense and sparse scenarios, and to detect potential new genes associated with diseases.

## Supplementary information

**Additional file 1** Additional results for simulation studies and schizophrenia data application. Results for GAW 17 data application.

## Data Availability

Datasets used in this paper are publicly available. R package for wAF method can be downloaded at https://github.com/songbiostat/wAF.

## Abbreviations

AF: Adaptive fisher
AR: Autoregressive
aSPU: Adaptive sum of powered score
BD: Bipolar disorder
CAST: Cohort allelic sums test
CMC: Combined multivariate and collapsing
dbGaP: Database of genotypes and phenotypes
GAIN: Genetic association information network
GHC: Generalized higher criticism
GLM: Generalized linear model
GWAS: Genome-wide association study
HC: Higher criticism
IMSGC: International multiple sclerosis genetics consortium
MAF: Minor allele frequency
MS: Multiple sclerosis
NGS: Next generation sequencing
RV: Rare variant
SCZ: Schizophrenia
SKAT: Sequence kernel association test
SNP: Single nucleotide polymorphism
SNV: Single nucleotide variant
SPU: Sum of powered score
SSU: Sum of squared score
TARV: Tree-based analysis of rare variants
UCSC: University of California, Santa Cruz
wAF: Weighted adaptive fisher

## Footnotes

1 Because of the logarithm, this MAF sampling algorithm often samples small MAFs and therefore yields more rare variants.
