# Supplementary material for: Adaptive Fisher method detects dense and sparse signals in association analysis of SNV sets
Source: BMC Med Genomics. 2020 Apr 3;13(Suppl 5):46. doi: 10.1186/s12920-020-0684-3 (PMC7118831; doi:10.1186/s12920-020-0684-3)
Supplement: Supplementary file 1 — Additional file 1 Additional results for simulation studies and schizophrenia data application. Results for GAW 17 data application. [file 12920_2020_684_MOESM1_ESM.pdf]

Supplementary

1 Simulation Studies

1.1 Type I errors

Table 1 show the empirical type I error rates of the six tests with a significance level of 0.05 for different the number of SNVs K.

Table S1: Empirical Type I Error Rates at the α Level of 0.05 with Continuous Trait

**K**	**wAF**	**wAF$_d$**	**aSPU**	**SKAT**	**SKAT-O**	**Min-P**
50	0.0466	0.0450	0.0438	0.0434	0.0426	0.0474
100	0.0510	0.0462	0.0456	0.0458	0.0482	0.0476
150	0.0492	0.0520	0.0480	0.0492	0.0512	0.0424
200	0.0584	0.0490	0.0604	0.0580	0.0588	0.0558
250	0.0476	0.0508	0.0466	0.0504	0.0488	0.0452
300	0.0446	0.0480	0.0474	0.0510	0.0518	0.0396
350	0.0464	0.0482	0.0436	0.0454	0.0468	0.0378
400	0.0492	0.0584	0.0500	0.0522	0.0544	0.0460
450	0.0534	0.0444	0.0522	0.0560	0.0578	0.0482
500	0.0498	0.0520	0.0504	0.0516	0.0576	0.0438

1.2 Power Curves

Figure 1 shows the power curves for six methods when all SNVs have effects of the same direction. Figure 2 and Figure 3 are simulated power curves with only rare variants: Figure 2 shows the results when SNVs have effects of varying directions, while Figure 3 shows the results when SNVs have effects of the same direction.

2 Real Data Application

2.1 Application to Schizophrenia Data

The GAIN SCZ data shows that wAF, $\mathrm{wAF_d}$ and aSPU have much smaller P-values than SKAT and SKAT-O on gene CERCAM. We calculate the P-values of two-sided score test on

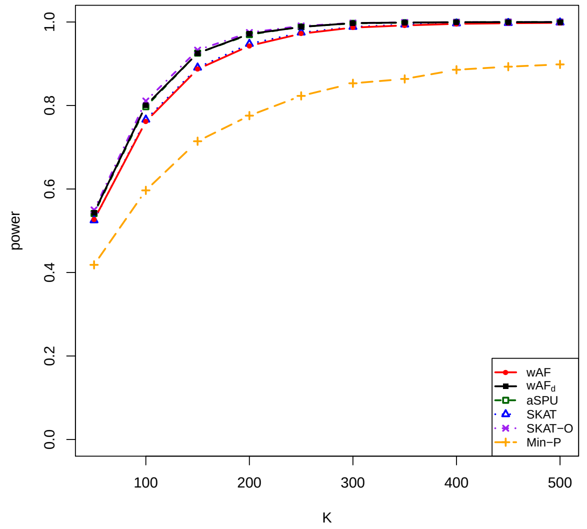

(a) Binary Trait, Dense Scenario

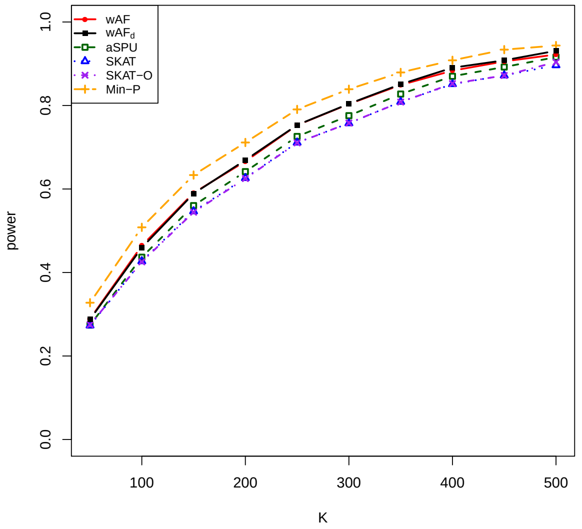

(b) Binary Trait, Sparse Scenario

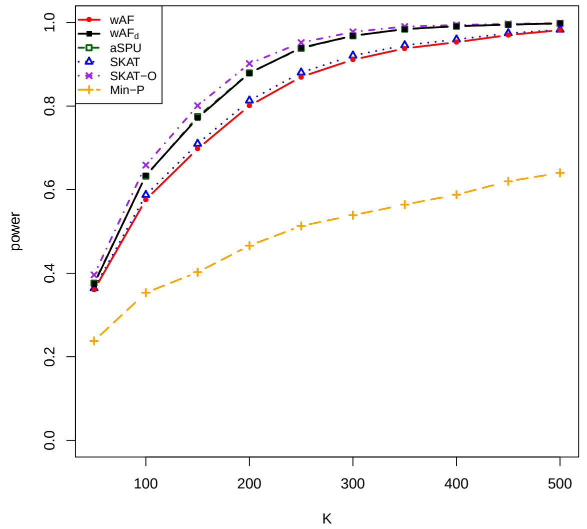

(c) Continuous Trait, Dense Scenario

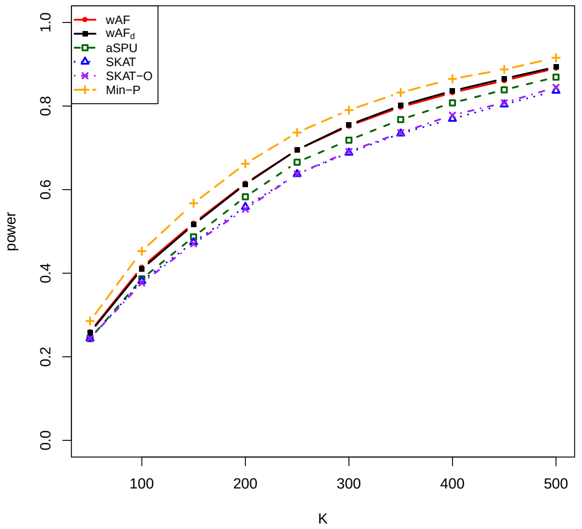

(d) Continuous Trait, Sparse Scenario

Figure S1: Comparison of empirical powers of six methods when SNV effects are of the same direction. (a) Power against varying number of loci K with binary trait in the dense scenario with effect proportion $\pi = 20\%$ and effect size $\delta = 0.25$. $K \in \{50, 100, ..., 450, 500\}$. (b) Power against varying number of loci K with binary trait in the dense scenario with effect proportion $\pi = 2\%$ and effect size $\delta = 1$. $K \in \{50, 100, ..., 450, 500\}$. (c) Power against varying number of loci K with continuous trait in the dense scenario with effect proportion $\pi = 20\%$ and effect size $\delta = 0.1$. $K \in \{50, 100, ..., 450, 500\}$. (d) Power against varying number of loci K with continuous trait in the dense scenario with effect proportion $\pi = 2\%$ and effect size $\delta = 0.5$. $K \in \{50, 100, ..., 450, 500\}$.

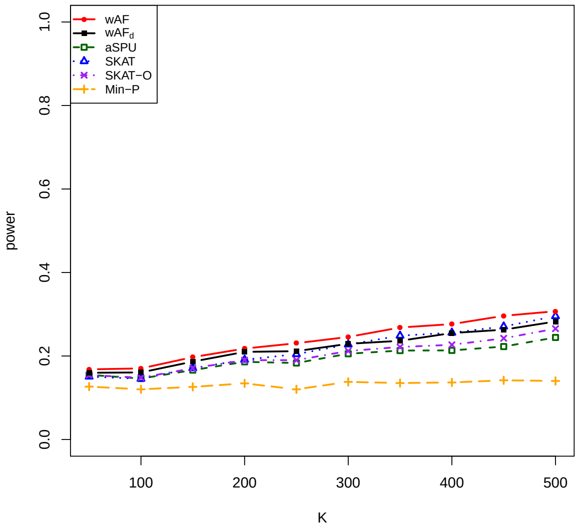

(a) Binary Trait, Dense Scenario

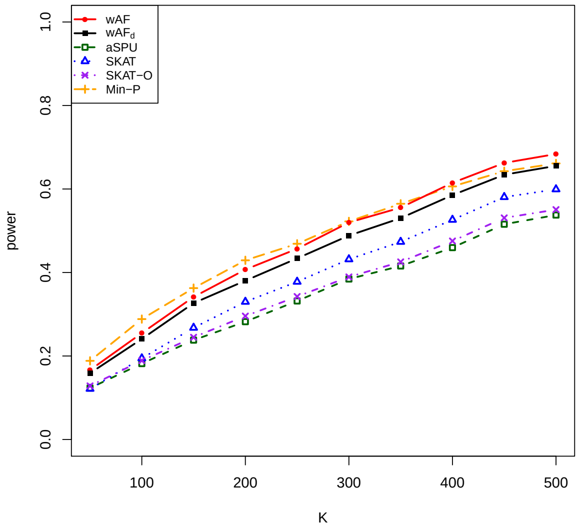

(b) Binary Trait, Sparse Scenario

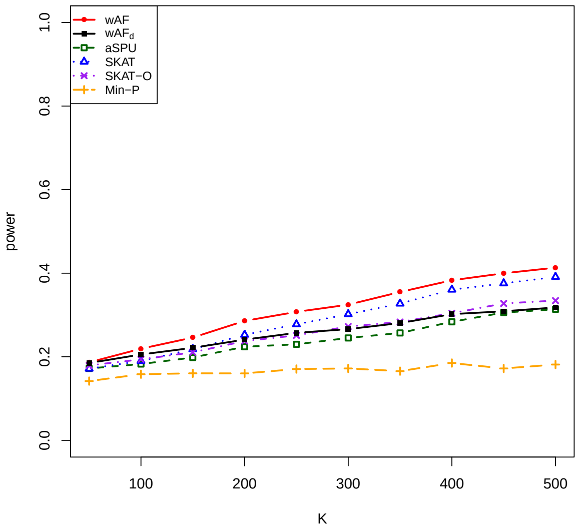

(c) Continuous Trait, Dense Scenario

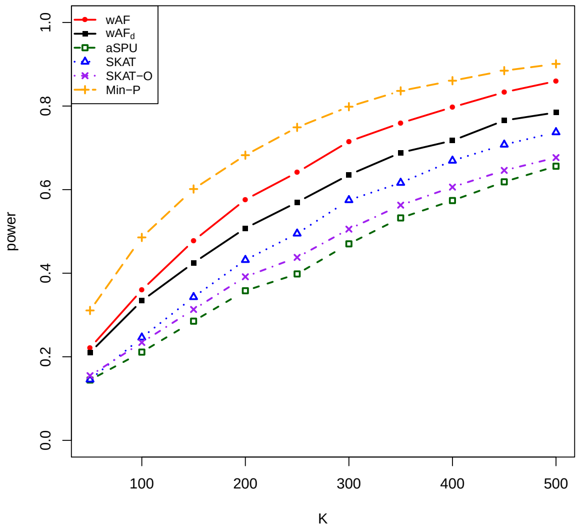

(d) Continuous Trait, Sparse Scenario

Figure S2: Comparison of empirical powers of six methods when SNV effects are of the same direction. (a) Power against varying number of loci K with binary trait in the dense scenario with effect proportion $\pi = 20\%$ and effect size $\delta = 0.5$. $K \in \{50, 100, ..., 450, 500\}$. (b) Power against varying number of loci K with binary trait in the dense scenario with effect proportion $\pi = 2\%$ and effect size $\delta = 2$. $K \in \{50, 100, ..., 450, 500\}$. (c) Power against varying number of loci K with continuous trait in the dense scenario with effect proportion $\pi = 20\%$ and effect size $\delta = 0.3$. $K \in \{50, 100, ..., 450, 500\}$. (d) Power against varying number of loci K with continuous trait in the dense scenario with effect proportion $\pi = 2\%$ and effect size $\delta = 1.5$. $K \in \{50, 100, ..., 450, 500\}$.

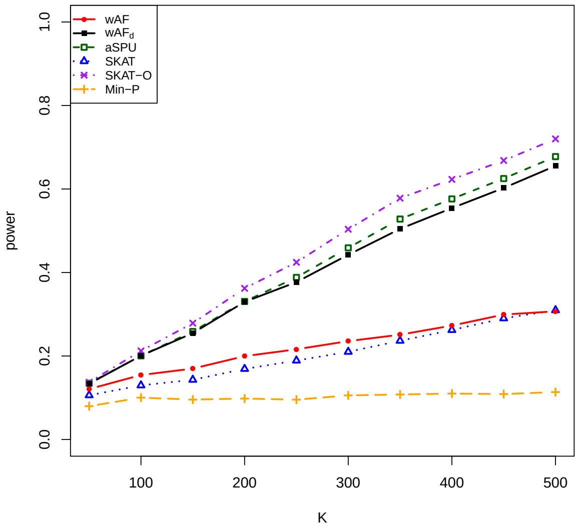

(a) Binary Trait, Dense Scenario

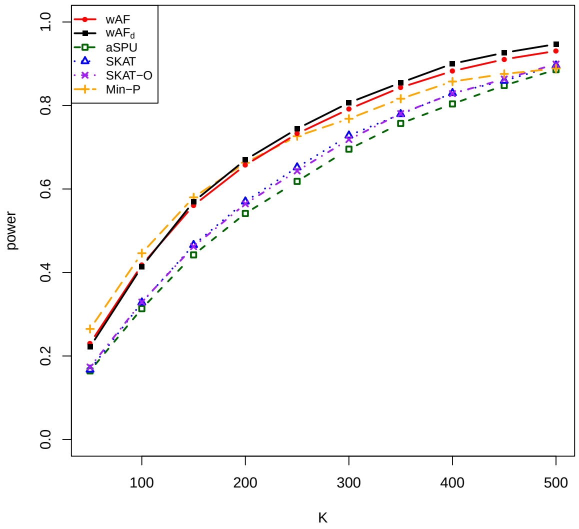

(b) Binary Trait, Sparse Scenario

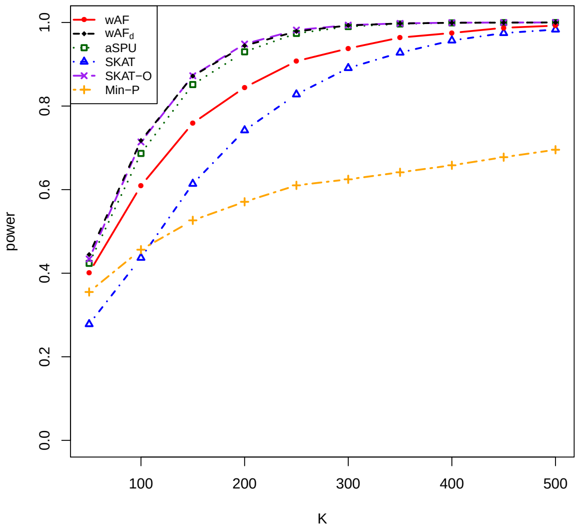

(c) Continuous Trait, Dense Scenario

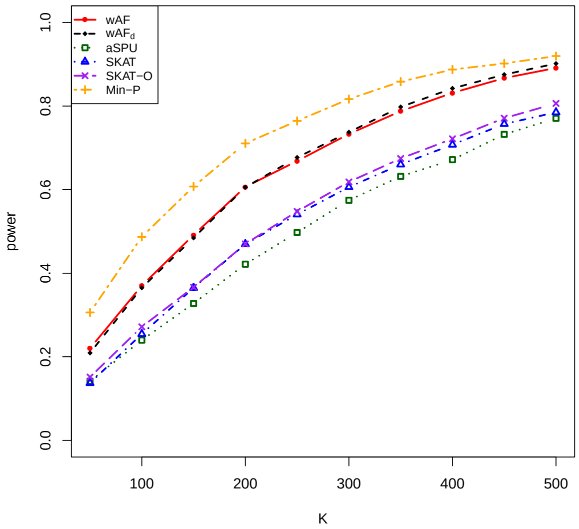

(d) Continuous Trait, Sparse Scenario

Figure S3: Comparison of empirical powers of six methods when SNV effects are of the same direction. (a) Power against varying number of loci K with binary trait in the dense scenario with effect proportion $\pi = 20\%$ and effect size $\delta = 0.3$. $K \in \{50, 100, ..., 450, 500\}$. (b) Power against varying number of loci K with binary trait in the dense scenario with effect proportion $\pi = 2\%$ and effect size $\delta = 2$. $K \in \{50, 100, ..., 450, 500\}$. (c) Power against varying number of loci K with continuous trait in the dense scenario with effect proportion $\pi = 20\%$ and effect size $\delta = 0.1$. $K \in \{50, 100, ..., 450, 500\}$. (d) Power against varying number of loci K with continuous trait in the dense scenario with effect proportion $\pi = 2\%$ and effect size $\delta = 1.5$. $K \in \{50, 100, ..., 450, 500\}$.

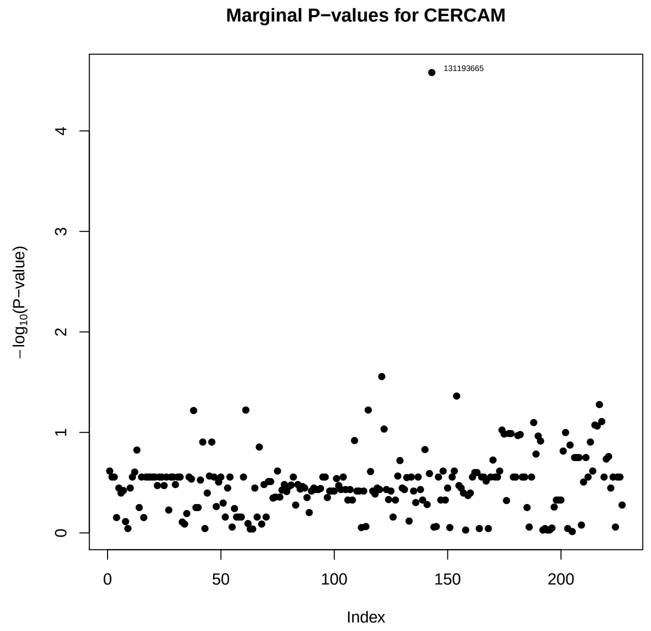

Figure S4: Score test P-values on each variant of CERCAM.

each variant of this gene, and find that SNP rs131193665 is much more significant than the others. This is a typical sparse scenario, in which wAF, wAF_d and aSPU are expected to perform better. Figure 4 gives the marginal P-values on negative log scale for each variant within CERCAM.

2.2 Application to GAW17 Data

In addition to the GAIN SCZ data, we also apply wAF, aSPU, SKAT, SKAT-O and Min-P to Genetic Analysis Workshop 17 (GAW17) mini-exome simulation data in Almasy et al. [2011]. In this dataset, 24, 487 SNVs in 3, 205 genes from 697 subjects are genotyped. If only rare variants (with MAFs no larger than 1%) are considered, there are 18, 131 rare variants in 2, 476 genes (that contain at least 1 rare variant). 200 sets of binary traits are simulated based on genotypes and three covariates: age, gender and smoking status. When only rare variants are considered, 35 causal genes have effects on the trait.

We follow the same procedure as Pan et al. [2014]. The six tests are applied on each of the 35 causal genes separately with a gene-wise significance level 0.05, and the corresponding powers are estimated using the 200 sets of phenotypes. Removing genes for which all tests have powers lower than 10%, the estimated powers for the remaining 17 genes are shown in Table S2. wAF gives the best power on eight genes (BCHE, VNN1, LPL, VLDLR, SIRT1, SOS2, HSP90AA1 and SREBF1), and has similar powers with the other methods on the other genes.

Table S2: Estimated Power for Some Causal Genes in GAW17 Data Anlysis

**Gene**	**wAF**	**aSPU**	**SKAT**	**SKAT-O**	**Min-P**
PIK3C2B	0.440	0.625	0.430	0.575	0.270
BCHE	0.245	0.210	0.155	0.145	0.165
KDR	0.360	0.295	0.385	0.400	0.050
VNN1	0.275	0.255	0.195	0.180	0.155
INSIG1	0.015	0.015	0.225	0.225	0.000
LPL	0.135	0.125	0.120	0.105	0.055
PTK2B	0.065	0.070	0.065	0.085	0.110
PLAT	0.145	0.145	0.120	0.180	0.060
VLDLR	0.110	0.085	0.100	0.085	0.090
SIRT1	0.110	0.090	0.095	0.090	0.035
VWF	0.060	0.020	0.015	0.030	0.135
FLT1	0.155	0.115	0.140	0.160	0.045
SOS2	0.270	0.220	0.270	0.210	0.100
HSP90AA1	0.345	0.145	0.255	0.190	0.290
SREBF1	0.105	0.075	0.075	0.070	0.105
PRKCA	0.030	0.030	0.185	0.185	0.000
RRAS	0.150	0.185	0.135	0.240	0.070

References

Laura Almasy, Thomas D Dyer, Juan Manuel Peralta, Jack W Kent, Jac C Charlesworth, Joanne E Curran, and John Blangero. Genetic analysis workshop 17 mini-exome simulation. In *BMC proceedings*, volume 5, page S2. BioMed Central, 2011.

Wei Pan, Junghi Kim, Yiwei Zhang, Xiaotong Shen, and Peng Wei. A powerful and adaptive association test for rare variants. *Genetics*, 197(4):1081–1095, 2014.
